# Change From 24-Hour Off-Site On-Calls to 12-Hour On-Site Shifts for Psychiatry SHOs Was Near-Unanimously Welcomed by SHOs, Senior Doctors and Allied Health Professionals in Cardiff

**DOI:** 10.1192/bjo.2024.489

**Published:** 2024-08-01

**Authors:** Charlotte Little, Vivek Majumder, Charles Pope

**Affiliations:** 1 Cardiff & Vale UHB, Cardiff, United Kingdom; 2 South West Yorkshire Foundation Trust, Wakefield, United Kingdom

## Abstract

**Aims:**

In January 2023, the rota for psychiatry SHOs within Cardiff and Vale UHB changed from 24-hour off-site on-call to 12-hour on-site shifts. This change occurred after rota gaps from sickness, increasing clinical pressures, and poor GMC survey feedback. We hypothesised that this would likely be received positively by SHOs, senior psychiatrists and other staff who work with the on-call SHO, and surveyed attitudes to the new rota. Our aim was to inform decisions about the rota going forward, and gather a baseline set of data for future comparison.

**Methods:**

Data were collected retrospectively, via three questionnaires created on Microsoft Forms and distributed by email to: junior doctors on the on-call rota (“SHOs”), registrars and consultant psychiatrists (“seniors”), and nurses and allied healthcare professionals (“AHPs”). We used a mixture of Likert scales and free-answer sections, surveying staff attitudes of the impact of the change in rota on Patient Safety, Workload, Training Impact, Working Relationships and Welfare. Questions were altered to suit the group being surveyed (e.g. AHPs were not asked to comment on welfare of SHOs).

Data were collected between the dates of 23/3/23 and 5/5/23.

**Results:**

A total of 63 respondents (17 SHOs, 12 seniors, 34 AHPs) completed the questionnaire.

Overall results were very positive in favour of the new rota, with a final overall question concluding that 100% SHOs preferred the new rota, as did 92% seniors and 92% AHPs. Other highlights include:

- 92% of all staff felt the on-call SHO could provide safer patient care, particularly at night.
- 82% SHOs and 83% seniors felt workload had improved or stayed the same.
- 71% SHOs had more training opportunities on-call (e.g. observing MHA assessments).
- 82% AHPs felt working relationships with SHOs had improved.
- 88% SHOs felt positive impact on their mental or physical wellbeing.

**Conclusion:**

The new rota was near-unanimously positively welcomed by each group of staff surveyed, in all domains studied.

These findings were presented to members of the clinical board, and used to justify continuing the rota in future. It has remained in-person since.

With baseline data gathered, we will repeat the survey in February 2024 to gain more data on current attitudes to the new rota, one year on from its implementation.

